# Micromorphological effect of calcium phosphate coating on compatibility of magnesium alloy with osteoblast

**DOI:** 10.1080/14686996.2016.1266238

**Published:** 2017-01-23

**Authors:** Sachiko Hiromoto, Tomohiko Yamazaki

**Affiliations:** ^a^ Corrosion Property Group, Research Center for Structural Materials, National Institute for Materials Science, Tsukuba, Japan; ^b^ Biosystem Control Group, Research Center for Functional Materials, National Institute for Materials Science, Tsukuba, Japan

**Keywords:** Magnesium, calcium phosphate coatings, topography, surface chemical property, cell adhesion, cell proliferation, biodegradable, 30 Bio-inspired and biomedical materials, 106 Metallic materials, 211 Scaffold / Tissue engineering / Drug delivery, 306 Thin film / Coatings

## Abstract

Octacalcium phosphate (OCP) and hydroxyapatite (HAp) coatings were developed to control the degradation speed and to improve the biocompatibility of biodegradable magnesium alloys. Osteoblast MG-63 was cultured directly on OCP- and HAp-coated Mg-3Al-1Zn (wt%, AZ31) alloy (OCP- and HAp-AZ31) to evaluate cell compatibility. Cell proliferation was remarkably improved with OCP and HAp coatings which reduced the corrosion and prevented the H_2_O_2_ generation on Mg alloy substrate. OCP-AZ31 showed sparse distribution of living cell colonies and dead cells. HAp-AZ31 showed dense and homogeneous distribution of living cells, with dead cells localized over and around corrosion pits, some of which were formed underneath the coating. These results demonstrated that cells were dead due to changes in the local environment, and it is necessary to evaluate the local biocompatibility of magnesium alloys. Cell density on HAp-AZ31 was higher than that on OCP-AZ31 although there was not a significant difference in the amount of Mg ions released in medium between OCP- and HAp-AZ31. The outer layer of OCP and HAp coatings consisted of plate-like crystal with a thickness of around 0.1 μm and rod-like crystals with a diameter of around 0.1 μm, respectively, which grew from a continuous inner layer. Osteoblasts formed focal contacts on the tips of plate-like OCP and rod-like HAp crystals, with heights of 2–5 μm. The spacing between OCP tips of 0.8–1.1 μm was wider than that between HAp tips of 0.2–0.3 μm. These results demonstrated that cell proliferation depended on the micromorphology of the coatings which governed spacing of focal contacts. Consequently, HAp coating is suitable for improving cell compatibility and bone-forming ability of the Mg alloy.

## Graphical Abstract

**Figure:**
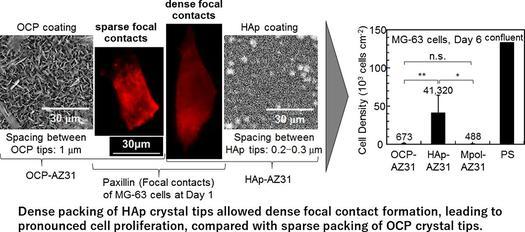

## 1. Introduction

Magnesium and its alloys have great potential for use as biodegradable/bioabsorbable metallic implant materials, owing to their high specific strength and having a similar Young’s modulus (41–45 GPa) to bone (3–20 GPa).[1–5] For the practical use of Mg/Mg alloys as biodegradable orthopedic devices, such as bone fixation screws, plates and nails, it is important to appropriately control the degradation (corrosion) speed and to improve the bone-tissue compatibility properties, such as bone conductivity and bone integration.[2,6,7] Rapid and unpredictable corrosion of Mg/Mg alloy implants can cause unexpected loss of mechanical integrity in the surrounding tissue. Rapid generation of corrosion products, such as H_2_ gas and OH^–^ and Mg^2+^ ions, can cause the formation of a gas cavity, inflammation and a severe foreign body response. Conversely, the pharmacological effect of Mg^2+^ ions is expected to promote healing of broken bone [8] or it may suppress inflammation.[9]

To appropriately control the corrosion speed of Mg/Mg alloys, various surface coatings have been developed, including phosphates, fluorides, calcium phosphates, bioglasses, and biodegradable polymers, using techniques such as anodization, electrodeposition, alkali heat treatments and sol-gel methods.[10–20] The formation of calcium phosphate (Ca-P) coatings for Mg/Mg alloys using electrodeposition techniques has been examined in numerous studies.[21–24] Ca-Ps, especially hydroxyapatite (HAp) and β-tricalcium phosphate (β-TCP), are often used for the coating of conventional Ti/Ti alloy implants and artificial bone to enhance their bone tissue compatibilities.[25] The Ca-P types formed on Mg/Mg alloy surfaces are dicalcium phosphate dihydrate (DCPD) or low crystalline HAp [10–13,21–24,26]; well-crystallized HAp is hardly formed in the presence of Mg^2+^ ions.[27,28] The interaction between Ca-P and bone tissues generally depends on the type of Ca-P and its surface topography [29–31]; however, the effect of these surface factors on cell adhesion, growth properties and cell viability have seldom been examined for Ca-P-coated Mg/Mg alloys.

We previously developed HAp and octacalcium phosphate (OCP) coatings for Mg/Mg alloys by a novel single-step chemical solution deposition method. [32–38] The type of Ca-P coating can be controlled with the pH of the treatment solution.[35,36] OCP and HAp coatings have two-layer structures: the inner layer is continuous and consists of nanocrystals, with a thickness of 1 to 5 μm depending on the treatment period; the inner layer of the OCP coating contains nanopores, with that of the HAp coating is microscopically dense.[35] Both types of Ca-P coatings exhibit protective effect in cell-culture medium and soft tissue of mouse. Both coatings suppressed subcutaneous inflammation and foreign body reaction in mouse by reducing the corrosion of substrate.[39] The protectiveness of OCP coating was slightly lower than that of HAp coating.[35,36] The difference in corrosion resistance is expected to affect cell behavior on OCP- and HAp-coated Mg/Mg alloys.

The outer layer consisted of highly crystalline plate-like OCP or rod-like HAp crystals, growing from the inner layer along the [002] axis of OCP and HAp crystals. Therefore, the edge of (001) or (010) OCP planes or the tip of (001) HAp plane is preferentially exposed to the surface. The length of OCP plates and HAp rods varied from about 0.5 to 5 μm with increasing treatment period. The width and thickness of OCP plates were about 1 μm and <30 nm, respectively. The diameter of HAp rods was about 150 nm for pure Mg treated at 363 K for 2 h.[35] As cell behavior depends on the surrounding topography [31,40], the crystal shape and size of HAp particles can affect the proliferation behavior of osteoblast-like cells.[30] Thus, the difference in morphology of OCP and HAp coatings could cause variances in cell adhesion and growth properties.

In this study, cell proliferation and cell adhesion behavior of OCP- and HAp-coated Mg alloy was examined using osteoblast-like MG-63 cells and AZ31 alloy.

## 2. Materials and methods

### 2.1. OCP and HAp coating of magnesium alloy

AZ31 disks of 15 mm diameter and 1 mm thickness were cut from extruded rods (Osaka Fuji Co., Amagasaki, Japan). The composition of the AZ31 rod is shown in Table 1. The surface of disks was ground with SiC papers (Buehler, IL, USA) up to #1200 and rinsed ultrasonically in acetone. Mechanically ground AZ31 disks were named Mpol-AZ31.

**Table 1.** Composition of AZ31 extruded rod (wt%).

Specimens	Al	Zn	Mn	Si	Cu	Ni	Fe	Mg	Extrusion ratio (–)
AZ31 extruded (15 mm^*φ*^)	2.89	1.01	0.43	–	<0.005	<0.005	<0.005	Balance	38

Coating treatment solutions were prepared with 500 mmol l^−1^ ethylenediaminetetraacetic acid (EDTA) calcium disodium salt hydrate (C_10_H_12_CaN_2_Na_2_O_8_, Ca-EDTA) solution, 500 mmol l^−1^ potassium dihydrogenphosphate (KH_2_PO_4_) solution, and 1 mol l^−1^ sodium hydroxide (NaOH) solution. The same volumes of the Ca-EDTA and KH_2_PO_4_ solutions were mixed and the pH was adjusted to 6.1 or 8.9 with the NaOH solution. Mpol-AZ31 disks were immersed in the treatment solutions at 90°C for 2 h. The pH of the solutions did not change after the treatment. OCP and HAp coatings were formed at pH 6.1 and 8.9, respectively. OCP- and HAp-coated AZ31 specimens were named OCP- and HAp-AZ31, respectively. The crystal structure was analyzed by X-ray diffraction (XRD) (RINT Ultima III, Rigaku, Tokyo, Japan). The surface and cross-sectional morphology of the coatings was observed by scanning electron microscope (SEM; FEI Quanta FEG250, OR, USA and Miniscope TM3000, Hitachi, Tokyo, Japan). Cross-section specimens were prepared by scraping off the OCP and HAp coatings with a cutter.

### 2.2. Measurement of surface pH and peroxide production

Surface pH of OCP- and HAp-AZ31 was measured using phenol red pH test paper (PR, pH 6.6–8.2, Advantec, Tokyo, Japan). A piece of pH test paper was placed on the surface immediately after a tiny amount of pure water was dropped on the surface. The surface pH was decided depending on the color of the pH test paper.

The peroxide production capacity of Mpol, OCP- and HAp-AZ31 surfaces was examined using semi-quantitative test paper (Quantofix® Peroxide 25, 0–25 mg l^−1^ H_2_O_2_, Machery-Nagel, Düren, Germany). Mpol-pure Mg and pure Ti (pure Mg and Ti disks were ground with SiC papers up to #1200), 2 mol l^−1^ MgCl_2_ solution and Mg(OH)_2_ suspension were used as references. The concentration of hydrogen peroxide (H_2_O_2_) produced on specimen surfaces was semi-quantified according to the graduation of blue color on the test paper. A piece of test paper was moistened with 10 μl of pure water, and then lightly pressed down on the sample surface for 10 s. The color of the test paper was observed.

### 2.3. Cell proliferation analysis by fluorescent staining

Human osteosarcoma cells MG-63 (cell no. RCB1890, RIKEN BioResource Center, Tsukuba, Japan) were used for cell proliferation analysis. Mpol-, OCP- and HAp-AZ31 disks were immersed in acetone for 10 s for sterilization and then dried in air. Then, the disks were placed in a 24-well plate (well diameter is about 16 mm). MG-63 cells were seeded into 24-well plates at a density of approximately 5 × 10^3^ cells per well, in minimum essential medium (MEM) supplemented with 10 vol.% fetal bovine serum (FBS), 100 U ml^−1^ penicillin, and 100 μg ml^–1^ streptomycin (Invitrogen, CA, USA).

**Figure 1.**
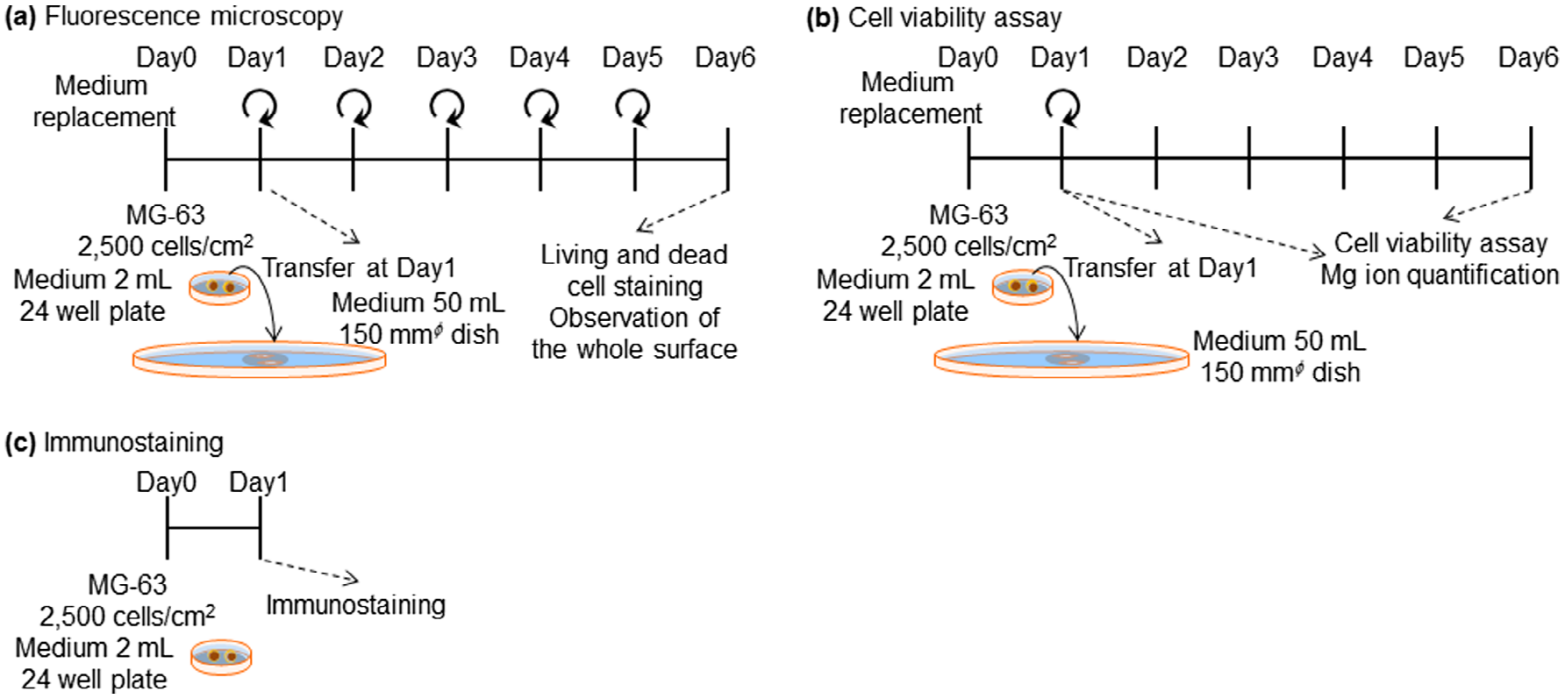
Experimental procedure for (a) fluorescence microscopy (b) cell viability assay, and (c) immunostaining of focal contacts.

After incubating at 37°C for 24 h in a 5% CO_2_ incubator, disks seeded with cells were each transferred into a *φ*15 cm dish and 50 ml of culture medium was added. Thereafter, medium was changed every 24 h. On days 2 and 6 of culture, the cytoplasm of living cells and the nuclei of dead cells were stained with calcein and propidium iodide (PI), respectively. The nuclei of living and dead cells were also stained with Hoechst33342. The cell culture procedure is shown in Figure 1(a).

Nine (3 × 3) images surveying the disk surface were taken by a fluorescence microscope (DMIL, Leica Microsystems, Wetzlar, Germany) with 1.25 × objective lens, following which they were merged to exhibit the entire surface of each disk. The surface of HAp-AZ31 after six days of culture was observed with an optical microscope and SEM.

### 2.4. Cell proliferation assay

The proliferation of MG-63 cells was measured using a luminescent cell viability assay kit (CellTiter-Glo™, Promega, WI, USA). Culturing of MG-63 cells on Mpol-, OCP- and HAp-AZ31 disks was performed in the same procedure as that for cell staining: except that medium-replacement was not performed after transferring the disks with cells to a *φ*15 cm dish at day 1 of culture as shown in Figure 1(b). At days 1 and 6 of culture, medium was removed from each well or dish, and cells were washed with 1 ml of phosphate buffered saline (PBS). Then, 0.5–1 ml of trypsin-EDTA (0.05%) (Gibco™, Thermo Fisher Scientific Inc., MA, USA) was added, and the 12-well plates or dishes were placed at 37°C for 5 min. After cells on the disks were detached, 0.5–1 ml of room temperature medium was added and cells were resuspended. Subsequently, the entire cell suspension was transferred to a new 24-well plate and incubated for 4.5 h.

CellTiter-Glo reagent was diluted twofold and 250 μl of the diluted reagent was added to each well after removing the medium. The 24-well plate was gently shaken for 2 min and then left standing at room temperature for 10 min. Then, 200 μl of the sample solution was transferred to a 96-well white solid plate, and luminescence was measured with micro-plate reader (MTP-880Lab, Corona, Ibaraki, Japan).

Magnesium ions in the used medium collected at days 1 and 6 were quantified by a colorimetric method using Xylidyl blue-I.[41,42]

### 2.5. Immunostaining of cells

MG-63 cells were cultured on OCP- and HAp-AZ31 using the same procedure described for the cell viability assay shown in Figure 1(c). At day 1 of culture, focal contacts and actin filaments were stained with paxillin and phalloidin, respectively, according to the protocol of the manufacturer. After medium was removed, 0.5 ml of 4 w/v% paraformaldehyde in PBS was added to each well, and cells were washed three times with 0.5 ml of PBS. Then, cells were incubated in 0.5 ml of PBS containing 1 w/v% bovine serum albumin (BSA) and 1 w/v% TritonX-100 on ice for 5 min, followed by washing with PBS. Subsequently, cells were incubated in 0.5 ml of 1 w/v% BSA in PBS on ice for 30 min. After the supernatant was removed, 50 μl of 1 μg ml^−1^ anti-paxillin antibody solution (Rabbit, H-114 sc-5574, Santa Cruz Biotechnology, Inc., TX, USA) was added to each well and incubated for 1 h at room temperature, away from light. After incubation, cells were washed with PBS, and 50 μl of secondary antibody solution (1 μg ml^−1^ Alexa Fluor 555 goat anti-rabbit IgG (H+L)) and 4 U ml^−1^ Alexa Fluor 488 phalloidin was added to each well and incubated for 1 h at room temperature, away from light. Afterwards, cells were washed with PBS, and 50 μl of 1 μg ml^−1^ Hoechst PBS solution was added and incubated for 15 min, following which they were washed with PBS. Antifade reagents was dropped on the surface of samples and a cover glass was placed on top for observation.

### 2.6. Statistical analysis

For cell proliferation tests, the results (cell number and Mg^2+^ ion concentration of the medium) were compared using a one-way analysis of variance (ANOVA) with Tukey-Kramer post hoc test for each incubation period. Statistical significance was set at *p* value less than 0.05.

## 3. Results

### 3.1. Morphology of OCP and HAp coating

**Figure 2.**
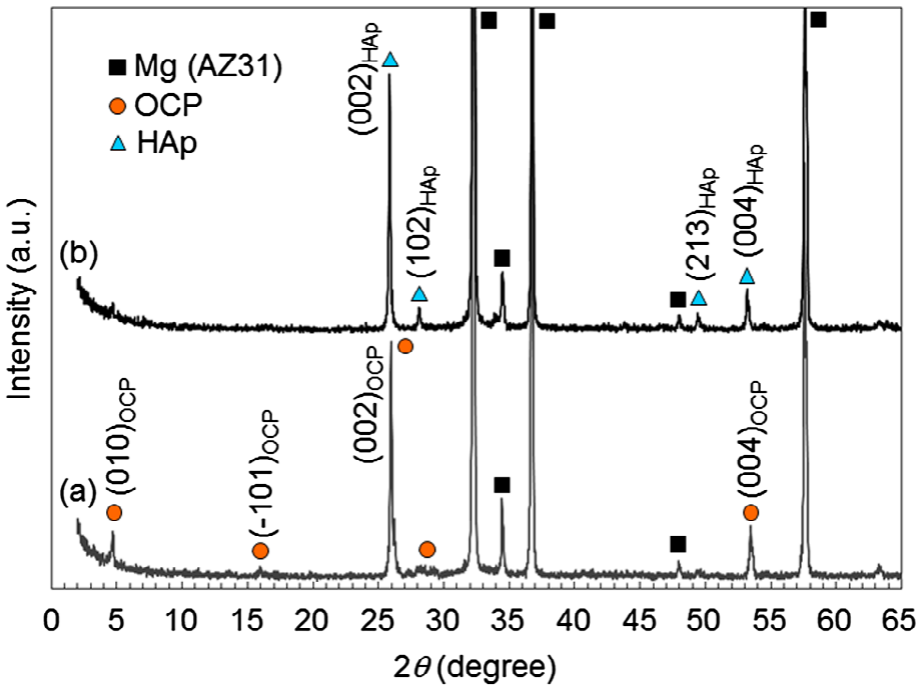
XRD patterns of OCP- and HAp-coated AZ31 alloy disks formed at (a) pH 6.1 and (b) pH 8.9, respectively. High relative intensities of (002) and (004) peaks indicates the preferential orientation of (002) planes of OCP and HAp crystals parallel to the substrate surface.

Figure 2 shows XRD patterns of AZ31 disks treated at pH 6.1 and 8.9. Diffraction peaks mainly from (002) and (004) planes of OCP and HAp were observed on pH 6.1- and pH 8.9-treated disks, respectively. This result agrees with the previous report.[36] The formation of OCP and HAp coatings on AZ31 disks was confirmed.

**Figure 3.**
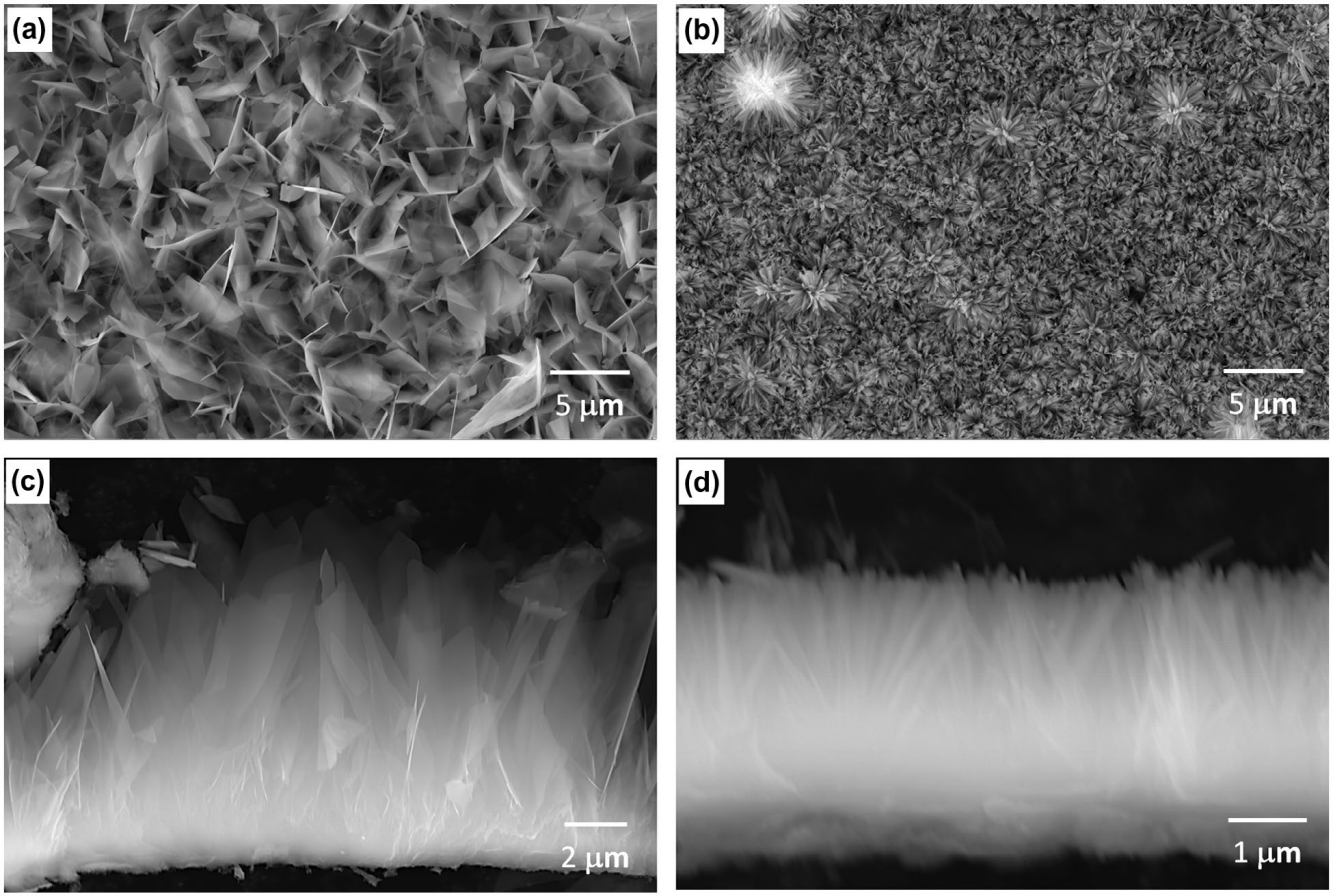
(a, b) Surface and (c, d) cross sectional SEM images of (a, c) OCP- and (b, d) HAp-AZ31. For cross-sectional observation, OCP and HAp coating layers were scraped off the surface of the substrate.

Figure 3 shows surface and cross-sectional SEM images of OCP and HAp coatings. The inner layers of the OCP and HAp coatings showed a similar thickness of 1.5–2 μm. The outer OCP layer consisted of thin plates, with dimensions of 3–5 μm length, 2–3 μm width and around 0.1 μm thickness, growing from the inner layer. The spacing between OCP crystal tips was 0.8–1.1 μm. The outer HAp layer consisted of thin HAp rods with a dimension of 2–3 μm length and around 0.1 μm in diameter, growing from the inner layer. The spacing between HAp crystal tips was 0.2–0.3 μm. HAp rods formed were denser than OCP plates.

### 3.2. Cell adhesion morphology on OCP- and HAp-AZ31

**Figure 4.**
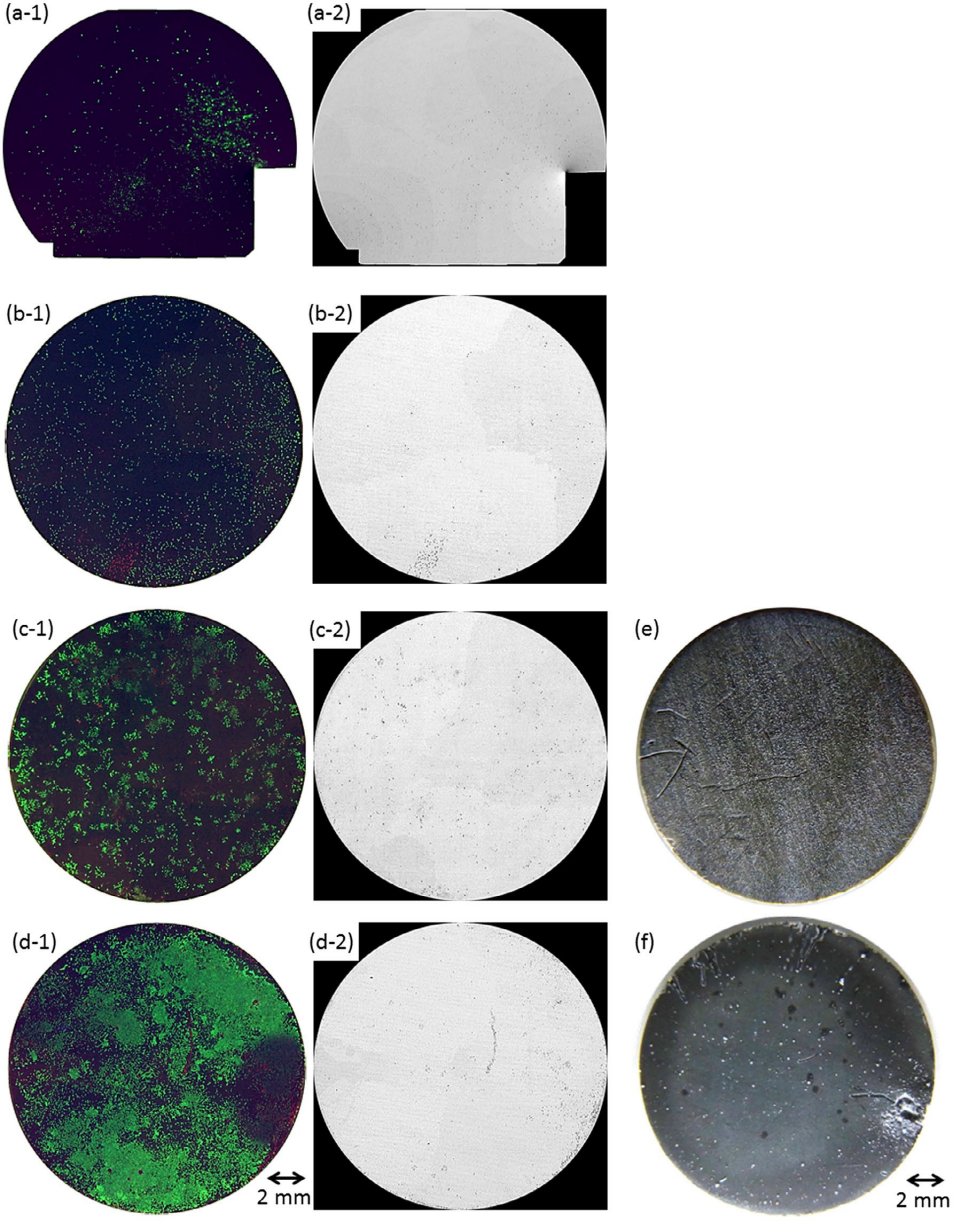
Fluorescence images of MG-63 cells on the entire surface of (a, c) OCP- and (b, d) HAp-AZ31, cultured for (a, b) two days and (c, d) six days. Optical images of (e) OCP- and (f) HAp-AZ31 disks after culturing cells for six days. (a-1–d-1) composite images of calcein-, PI-, and Hoechst33342-stained cell images (living cells, green; dead cells, red; nuclei of both cells, blue), and (a-2–d-2) PI-stained images converted to monochrome image (dead cells, black).

Figure 4(a-1)–(d-1) shows composite fluorescence images of living and dead cells and nuclei of both cells on the entire surface of OCP- and HAp-AZ31 at days 2 and 6. Figure 4(a-2)–(d-2) shows dead-cell images converted to monochrome. Figure 4(e) and (f) shows optical microscopic images of the entire surface at day 6. Fluorescence image of Mpol-AZ31 could not be taken because all of the cells were dead and detached from the surface. HAp-AZ31 showed higher living cell density than OCP-AZ31 at days 2 and 6.

At day 2, HAp-AZ31 exhibited a more homogeneous distribution of living cells than OCP-AZ31, on which living cells were sparsely distributed. At day 6, cells had almost completely covered the HAp-AZ31 surface, while cells on OCP-AZ31 surfaces formed homogeneously distributed colonies. Dead cells on HAp-AZ31 were obviously localized, while dead cells on OCP-AZ31 were almost homogeneously distributed amongst the living cells at days 2 and 6.

OCP- and HAp-AZ31 showed differences in corrosion morphology. OCP-AZ31 exhibited homogeneously distributed micro-pits with white corrosion products as shown in Figure 4(e). HAp-AZ31 exhibited localized distribution of dead cell regions, some of which associated with visible filiform and round corrosion pits (Figure 4(f)).

**Figure 5.**
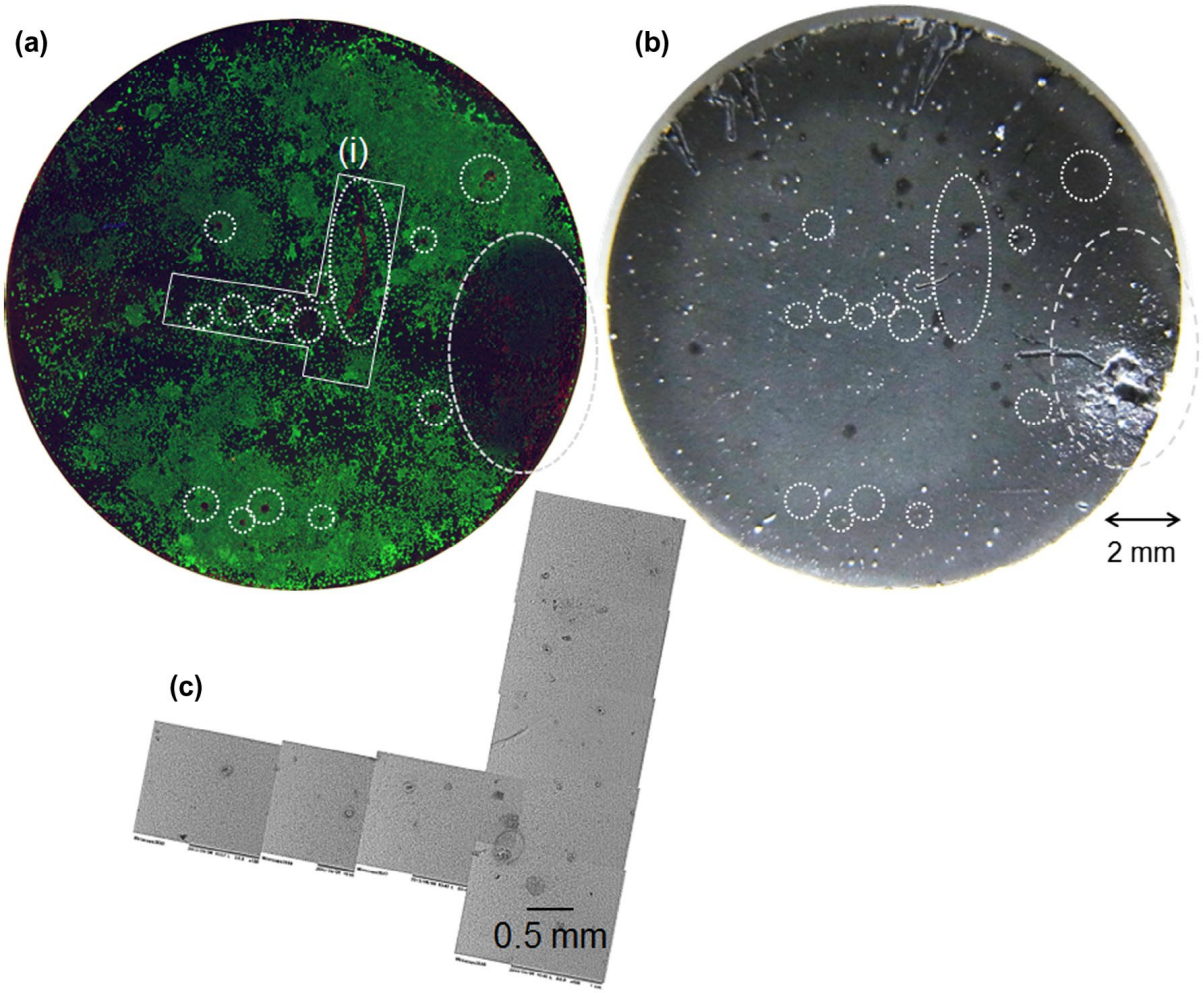
(a) Composite image of calcein-, PI-, and Hoechst33342-stained cell images (living cells, green; dead cells, red; nuclei of both cells, blue) and (b) optical images of the entire surface of HAp-AZ31 at day 6 and (c) SEM image of dead cell regions (i) on image (a). Dead-cell regions are encircled with dotted lines on images (a) and (b).

To examine the relationship between corrosion and cell-death on HAp-AZ31, magnified fluorescence and optical microscopic images, and a SEM image of an area containing dead-cell regions were examined: these are shown in Figure 5. Obvious dead cell regions are encircled with dotted line on the fluorescence and optical microscopy images. Scratches in the upper side of the disk were formed with a pair of tweezers, which was used to remove the disk from the cell-culture plate. The largest dead-cell region on the right side of the disk corresponds to the largest filiform corrosion. Relatively small dead-cell regions sometimes corresponded to visible micro-pits with a diameter of around 100 μm, but did not always correspond to visible corrosion pits. Even with a scanning electron microscope, traces of visible corrosion were not observed on the surface of some dead-cell regions.

### 3.3. Cell proliferation on OCP- and HAp-AZ31

**Figure 6.**
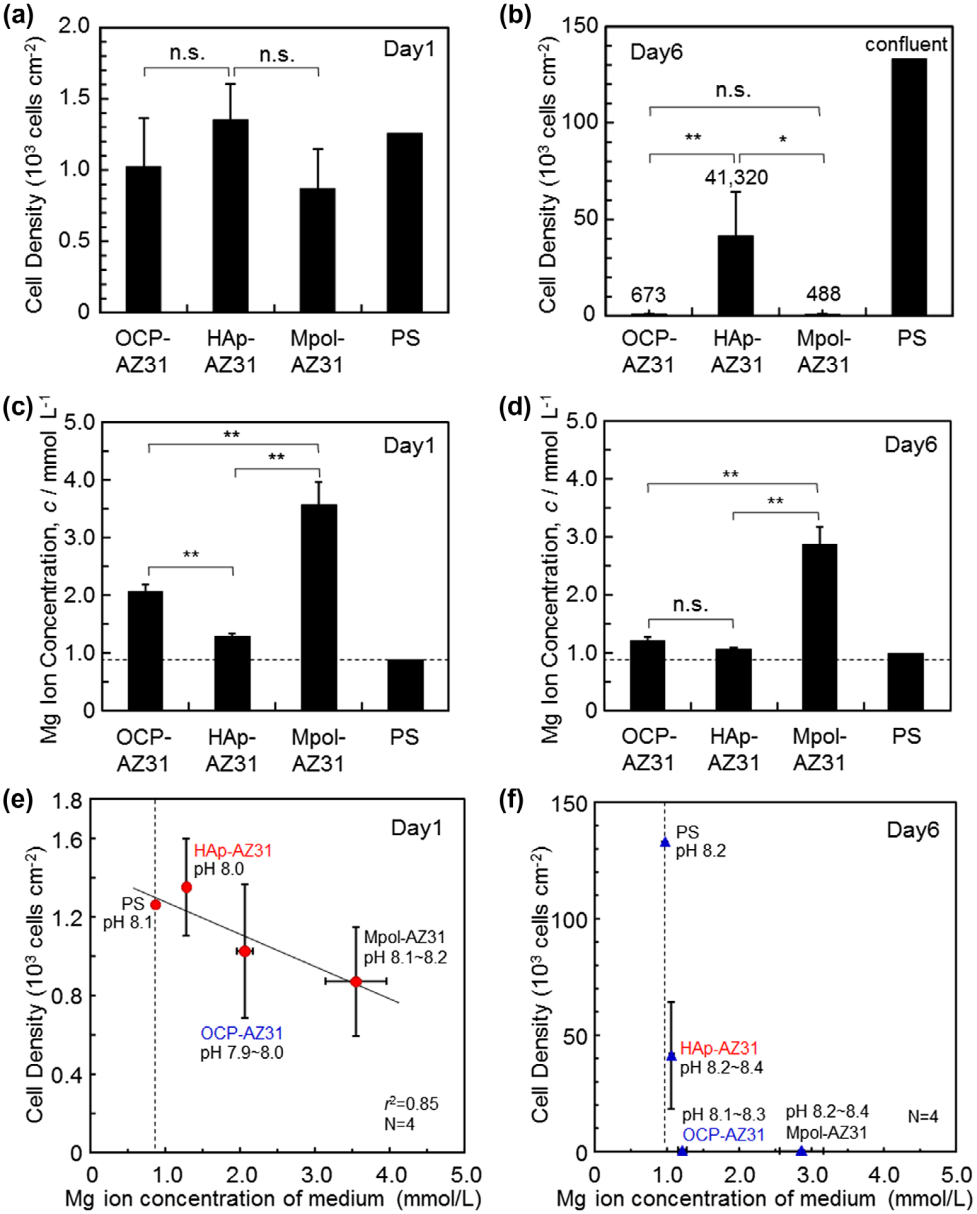
(a, b) Cell densities on OCP-, HAp- and Mpol-AZ31 and polystyrene (PS) dish at days 1 and 6. (c, d) Magnesium ion concentration of medium at days 1 and 6 used for cell culture on OCP-, HAp- and Mpol-AZ31 and PS. Dashed lines in (c) and (d) correspond to the original Mg^2+^ ion concentration of the medium. Cell density as a function of Mg^2+^ ion concentration in the medium at (e) day 1 and (f) day 6.

Cell density was obtained by counting cells removed from OCP-, HAp- and Mpol-AZ31 and polystyrene dishes (PS) at days 1 and 6, which are shown in Figure 6(a) and (b). The obtained cell densities might be slightly lower than the actual values because some cells presumably remained attached to the specimen surfaces. At day 1, there was no statistically significant difference in cell density between specimens; however, OCP- and HAp-AZ31 showed slightly higher cell densities than Mpol-AZ31, and HAp-AZ31 had a slightly higher cell density than OCP-AZ31. At day 6, HAp-AZ31 had significantly higher cell density than OCP- and Mpol-AZ31 surfaces. Cells proliferated about by 30 times on HAp-AZ31 from day 1 to day 6, whereas cell growth on OCP- and Mpol-AZ31 surfaces was remarkably suppressed.

Magnesium ion concentrations of media collected at days 1 and 6 are shown in Figure 6(c) and (d). Since the whole medium was refreshed at day 1, the value at day 6 is the accumulation of Mg^2+^ ions released from days 2 to 6. OCP and HAp coatings significantly suppressed Mg^2+^ ion release from the AZ31 substrate at day 1. The suppression effect of the HAp coating was more pronounced than that of the OCP coating. The release rate was reduced after day 1. OCP- and HAp-AZ31 specimens showed slight Mg^2+^ ion release, and Mpol-AZ31 showed significant Mg^2+^ ion release, at day 6.

Cell density was plotted as a function of the Mg^2+^ ion concentration, as shown in Figure 6(e) and (f). At day 1, there was a certain correlation between cell density and Mg^2+^ ion concentration, as the cell density decreased with a corresponding increase in Mg^2+^ ion concentration. At day 6, cell density on OCP-AZ31 became drastically lower than that on HAp-AZ31, although there was no statistically significant difference in Mg^2+^ ion concentration. These results suggest that the initial corrosion of the AZ31 substrate slightly affected the initial cell survival and/or cell adhesion. The subsequent corrosion with lower corrosion rate did not dominate the cell growth.

**Figure 7.**
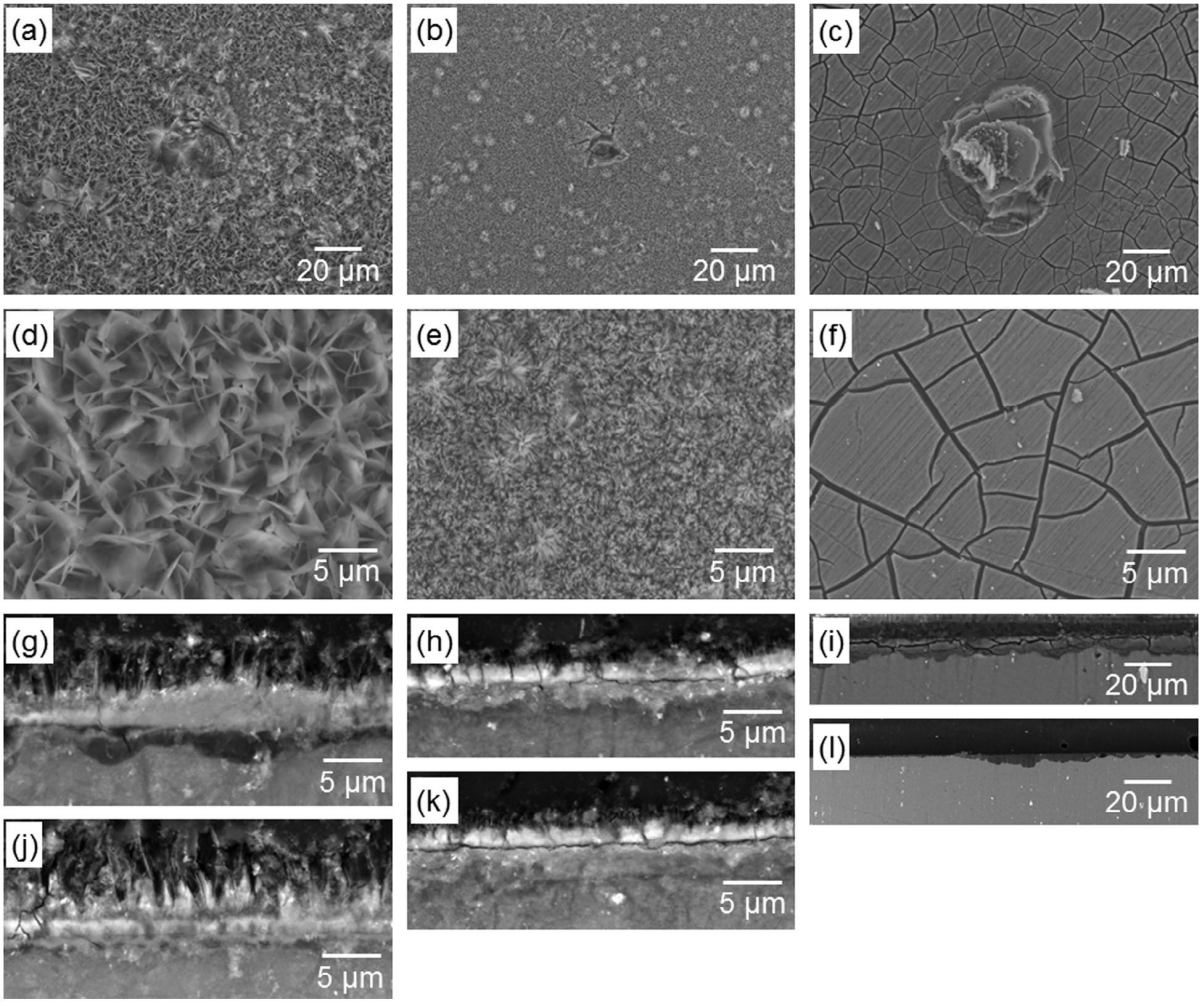
(a–f) Surface and (g–l) cross-sectional SEM images of (a, d, g, j) OCP-, (b, e, h, k) HAp- and (c, f, i, l) Mpol-AZ31 after six days culture of MG-63 cells. Surface images (a–c) with a typical pit and (d–f) without apparent corrosion. Cross-sectional images (g–i) with a relatively thick corrosion layer and (j–l) without obvious corrosion layer.

Figure 7 shows surface and cross-sectional SEM images of the specimens after six days of culture. Low magnification surface images (Figure 7(a)–(c)) show that pits with diameters of 20–30 μm were formed on all surfaces. High magnification surface images (Figure 7(d)–(f)) show that the original morphology of OCP and HAp coatings was well maintained after culturing. Mpol-AZ31 was covered with and precipitates from medium, which according to a previous study, consisted of calcium phosphate and Mg(OH)_2_.[39] Under the coatings, OCP-AZ31 locally showed a dark gray layer of corrosion product of Mg(OH)_2_, with a thickness of 2–4 μm (Figure 7(g) and (j)). HAp-AZ31 had a trace layer of corrosion product (Figure 7(h)). Mpol-AZ31 had a layer of corrosion product with a thickness of 10–50 μm (Figure 7(i) and (l)). The smaller formation of corrosion products underneath the HAp coating than those formed underneath the OCP coating corresponds to the lower Mg^2+^ ion concentration of HAp-AZ31 compared with OCP-AZ31 (Figure 6(c) and (d)).

### 3.4. Cell adhesion behavior on OCP- and HAp-AZ31

**Figure 8.**
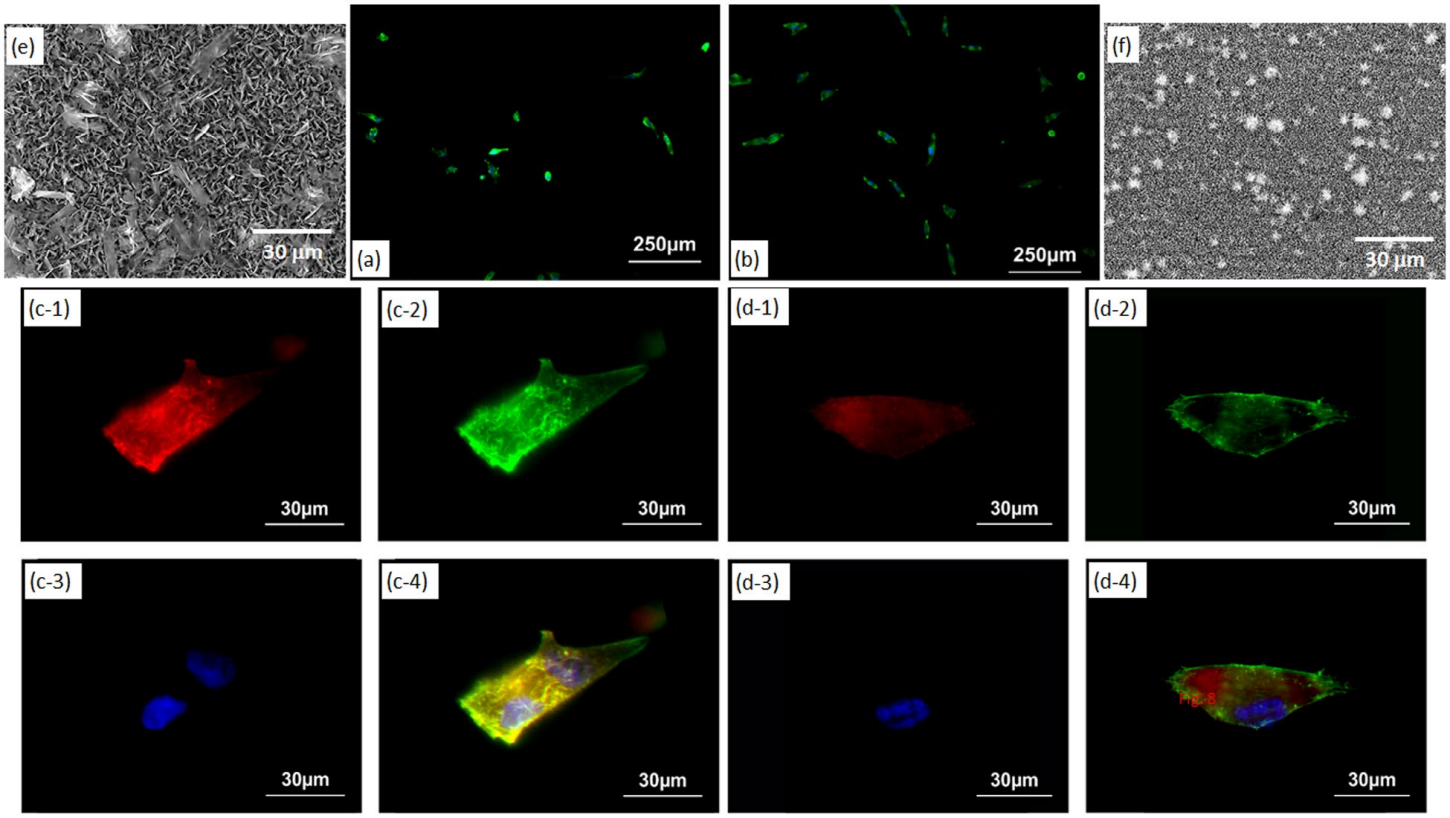
Morphology of MG-63 cells on (a) OCP- and (b) HAp-AZ31 at day 1. Focal contact formation of MG-63 cell on (c) OCP- and (d) HAp-AZ31. (c-1, d-1) Anti-paxillin-stained images (focal contact, red), (c-2, d-2) phalloidin-stained images (actin filament, green), (c-3, d-3) DAPI-stained images (nucleus, blue), and (c-4, d-4) composite images of anti-paxillin-, phalloidin- and DAPI-stained images. Surface SEM images of (e) OCP- and (f) HAp-AZ31, with the same magnification as the cell images (c) and (d).

Figure 8(a) and (b) show fluorescence microscopy images of MG-63 cells cultured for one day on OCP- and HAp-AZ31, respectively. Cell size on HAp-AZ31 was on an average larger than that on OCP-AZ31. Cells on HAp-AZ31 were more elongated than those on OCP-AZ31. Figure 8(c) and (d) show fluorescence microscopy images of focal contacts, actin filaments, nuclei, and a merged image of a cell on OCP- and HAp-AZ31, respectively. Figure 8(e) and (f) show surface SEM images of as-prepared OCP- and HAp-AZ31, with the same magnification as that of the fluorescence images. The shape and density of focal contacts formed on OCP-AZ31 were similar to those of tips of plate-like OCP crystals, and actin filaments preferentially distributed on focal contacts. Dot-shaped focal contacts were distributed as densely and homogeneously as tips of rod-like HAp crystals on HAp-AZ31, and actin filaments ran along the longitudinal axis of the cell body. The density of focal contacts appeared to be lower on OCP-AZ31 than that on HAp-AZ31, which is presumably attributed to the wider spacing between crystal tips on OCP-AZ31 than those on HAp-AZ31: 0.8–1.1 μm and 0.2–0.3 μm, respectively. The morphologies of focal contacts and actin filaments were also observed at day 3, with similar results being obtained.

**Figure 9.**
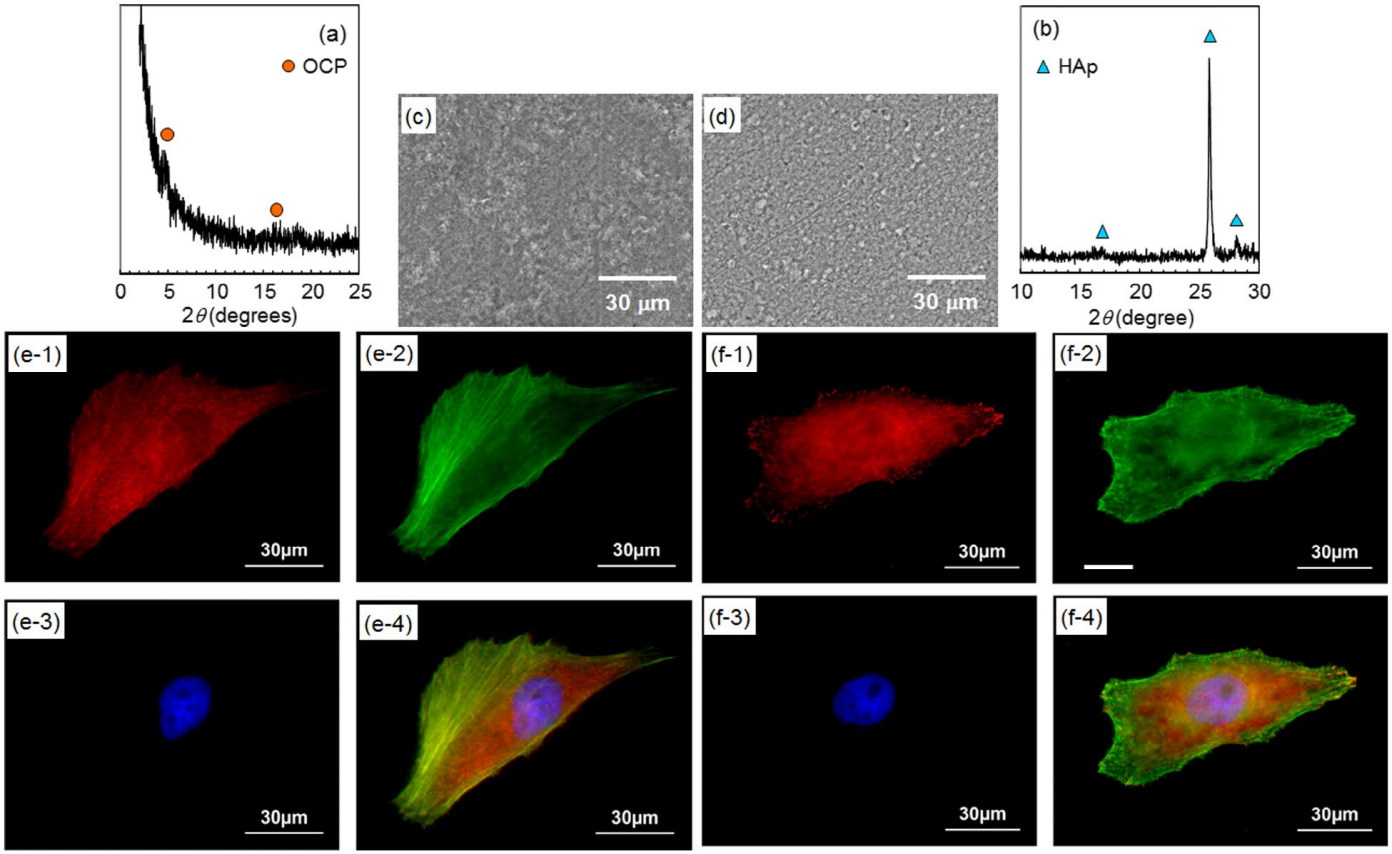
(a, b) XRD patterns and (c, d) SEM images of outer layer removed (a, c) OCP- and (b, d) HAp-AZ31. Focal contact formation of MG-63 cells on outer layer removed (e) OCP- and (f) HAp-AZ31. Plate-like and rod-like crystals were polished off surface with 6-μm-diamond paste. (e-1, f-1) Anti-paxillin-stained images (focal contact, red), (e-2, f-2) phalloidin-stained images (actin filament, green), (e-3, f-3) DAPI-stained images (nucleus, blue), and (e-4, f-4) composite images of anti-paxillin-, phalloidin- and DAPI-stained images.

After plate-like OCP and rod-like HAp crystals were polished off surfaces with diamond paste, the morphologies of focal contacts and actin filaments of MG-63 cells were observed. The remaining inner continuous layer was confirmed by XRD measurement (Figure 9(a) and (b)) and SEM observation (Figure 9(c) and (d)). Figure 9(e) and (f) show fluorescence microscopy images of focal contacts, actin filaments, nuclei, and a merged image of a cell cultured for one day on OCP- and HAp-AZ31, without plate-like or rod-like crystals. In both cases, focal contacts were homogeneously distributed in the cell body, while actin filaments preferentially ran along the longitudinal axis of the cell body. It was demonstrated that the localization of focal contacts disappeared after removing plate-like crystals on OCP-AZ31.

### 3.5. Surface chemical properties of OCP and HAp coatings

**Figure 10.**
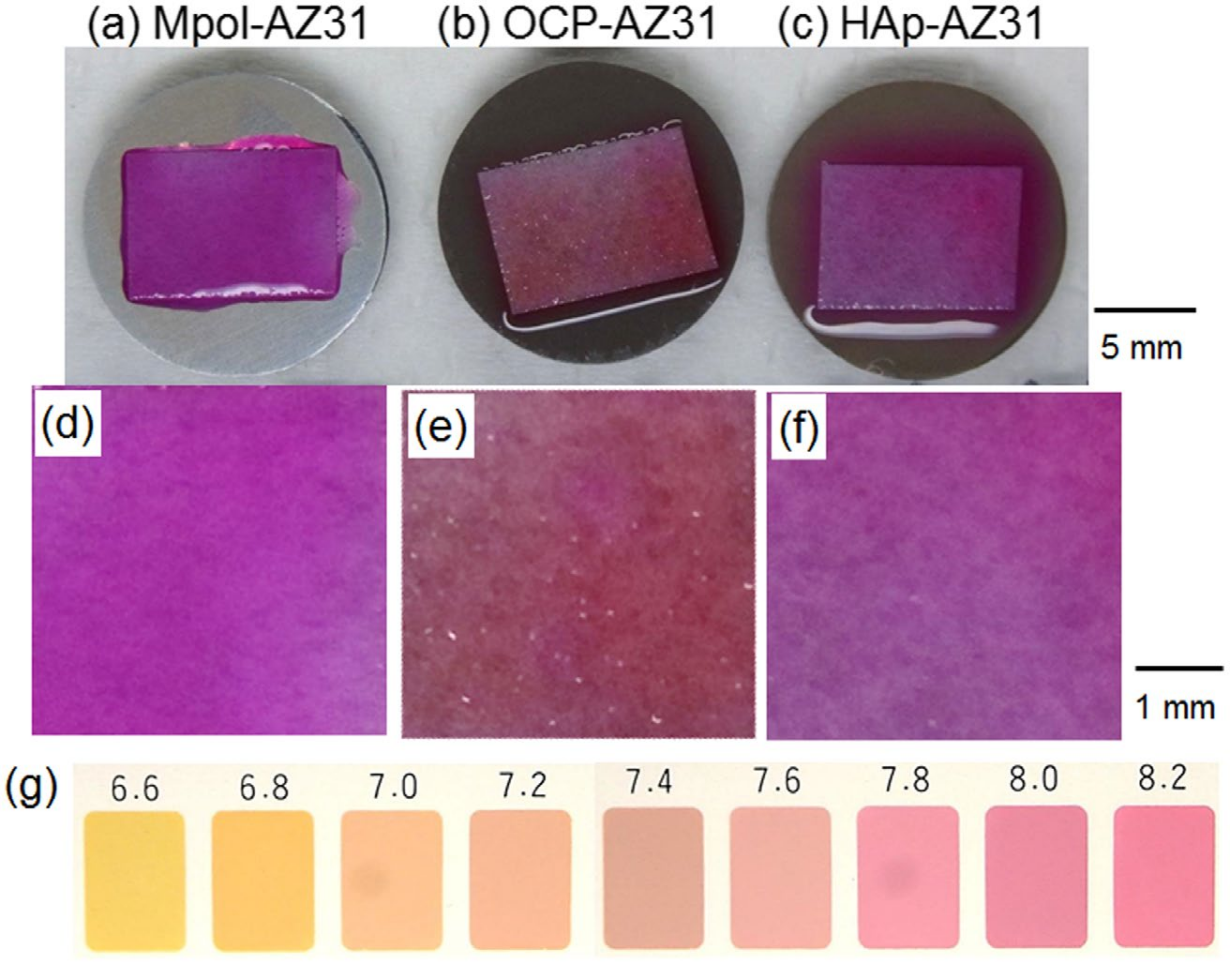
Optical images of the entire area of (a) Mpol-, (b) OCP- and (c) HAp-AZ31 disks, on which a piece of wet pH paper was placed for 30 min. Magnified optical images of pH paper placed on (d) Mpol-, (e) OCP- and (f) HAp-AZ31. (g) Color samples at various pH values.

Figure 10 shows optical images of a piece of pH paper placed on Mpol-, OCP- and HAp-AZ31. The pH of OCP and HAp coatings was around 7.4 and greater than 8.2, respectively. The OCP coating had a lower pH than the HAp coating.

**Figure 11.**
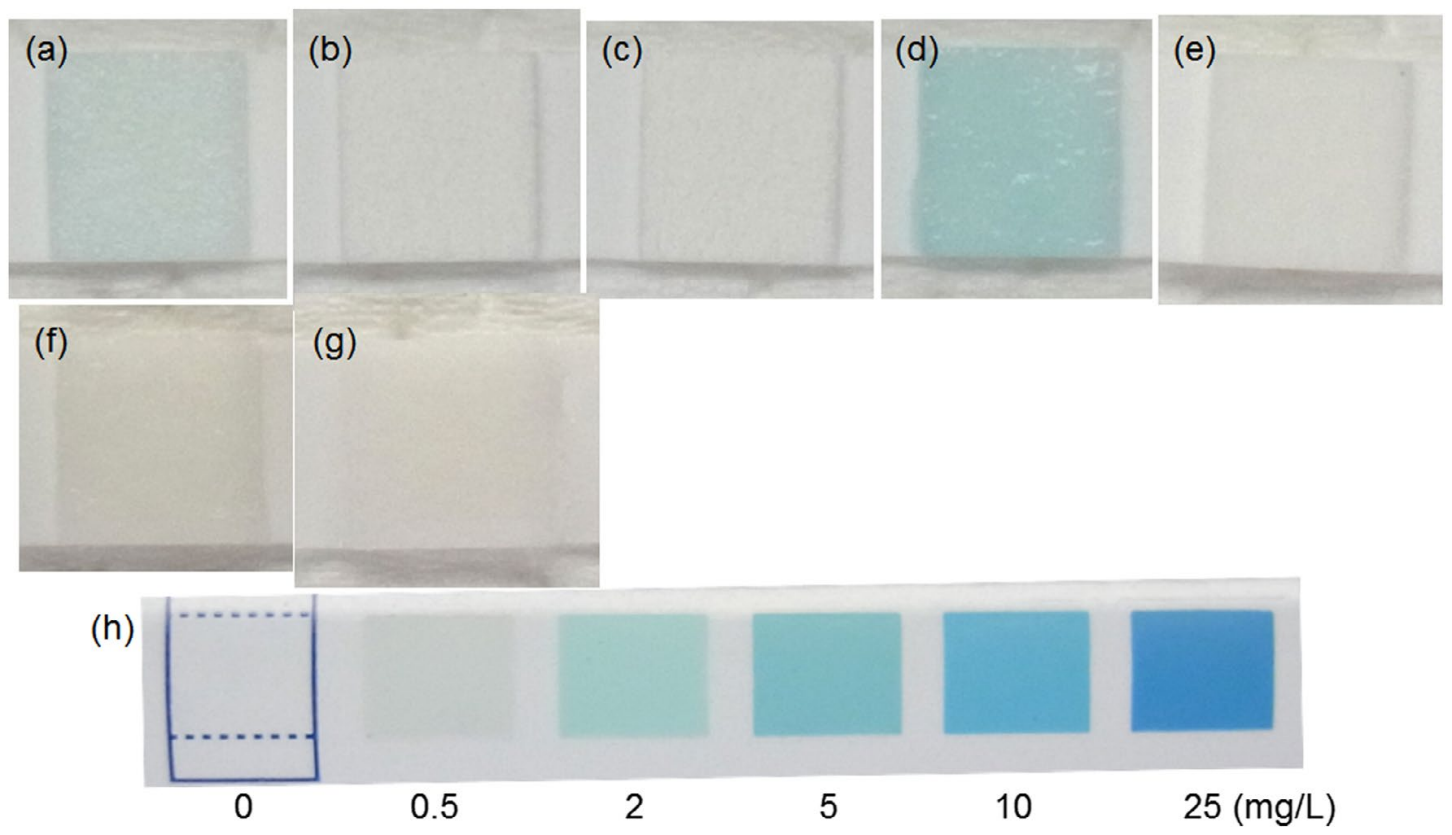
Optical images of a piece of H_2_O_2_ semi-quantitative test paper placed on the surface of (a) Mpol-, (b) OCP- and (c) HAp-AZ31, (d) Mpol-pure Mg and (e) Mpol-pure Ti. Optical images of a test paper moistened with (f) 2 mol l^−1^ MgCl_2_ solution and (g) Mg(OH)_2_ suspension. (h) Color samples at various H_2_O_2_ concentrations.

Because it was reported that MgO and ZnO powders generated reactive oxygen species and exhibited antibacterial activity against Gram-positive bacteria [43] and that galvanically coupled Mg–Ti particles generated reactive oxygen species which exhibited a killing capability of MC3T3-E1 cells [44], H_2_O_2_ generation on the specimens was examined. Figure 11 shows optical images of a piece of wet H_2_O_2_ test paper placed on various specimens. The test papers for Mpol-AZ31 and pure Mg exhibited an existence of about 0.5 and 2.0 mg l^−1^ of H_2_O_2_, respectively. In contrast, the test papers for OCP- and HAp-AZ31 was not colored. No generation of H_2_O_2_ in MgCl_2_ solution and Mg(OH)_2_ suspension indicates that Mg^2+^ ions did not cause coloring of the H_2_O_2_ test paper. It was revealed that H_2_O_2_ was generated from AZ31 and pure Mg surfaces and that OCP and HAp coatings suppressed H_2_O_2_ generation.

## 4. Discussion

### 4.1. Adhesion and proliferation of MG-63 cells on OCP- and HAp-AZ31

OCP and HAp coatings improved MG-63 cell proliferation on AZ31 alloy by reducing the corrosion of the substrate alloy (Figures 4 and 6). The improvement of cell proliferation with the HAp coating was macroscopically more pronounced than that with the OCP coating. The higher proliferation on the HAp coating is attributed to the smaller release of Mg^2+^ ions, and the denser packing of crystal tips for HAp-AZ31 than those for OCP-AZ31 (Figures 6 and 8) as mentioned in Section 4.2. However, such a difference in cell proliferation would disappear for a long period of *in vivo* implantation time: only a slight difference was observed between OCP- and HAp-AZ31 in the thickness of the fibrous tissue layer formed due to foreign body reaction (Supplemental Figure S2). This is attributed to the disappearance of OCP plate-like crystals in 16 weeks of implantation (Supplemental Figure S3).[39]

The relatively alkaline property of the HAp coating (Figure 10) seems to have no effect on cell adhesion or proliferation. Prevention of H_2_O_2_ generation with OCP and HAp coatings (Figure 11) allowed discussion of the morphological effect of the coatings on cell behavior. The H_2_O_2_ generation on Mpol-AZ31 presumably played an important role in the cell compatibility. However, further investigation is necessary to elucidate the effect of surface pH and H_2_O_2_ generation on cells on Mg alloy surfaces.

Cells around obvious corrosion pits were selectively dead on HAp-AZ31 surfaces (Figure 4). Corrosion of Mg alloy generates mainly Mg^2+^ and OH^–^ ions and H_2_ gas, and a part of Mg^2+^ and OH^–^ ions deposited as Mg(OH)_2_.[39] Seuss et al. [45] reported that a pH increase of medium reduced the proliferation of Hela cells.^[45]^ Formation of H_2_ gas bubbles and Mg(OH)_2_ gel around cells might separate the cell bodies from medium and prevent the cell metabolism. The cell death was thus attributed to a pH increase and H_2_ and Mg(OH)_2_ generation caused by corrosion. In contrast, cells were also dead in the regions without microscopically visible corrosion pits (Figures 3 and 4). In this situation, local corrosion underneath OCP and HAp coatings (Figure 7) could occur and H_2_ gas and Mg^2+^ and OH^–^ ions could permeate through the coating layer, leading to cell death. Further investigation is necessary to identify the dominant factor of local cell death.

Corrosion on HAp-AZ31 was remarkably localized, compared with that on OCP-AZ31 (Figure 4(e) and (f)), and caused the localized cell death on HAp-AZ31 (Figure 4(c) and (d)). The corrosion morphology was attributed to the microstructure of the inner continuous layer of the coatings: OCP and HAp coatings showed nanopores and microscopically dense structure, respectively.[35] On the other hand, this difference in corrosion morphology was not observed after a long period of *in vivo* implantation. Both OCP- and HAp-AZ31 showed similar large round pits in subcutaneous tissue of mouse after 16 weeks of implantation .[39] This was attributed to the low diffusivity in soft tissue compared with that in bulk medium. The presence of immune cells like macrophages might also influence the corrosion morphology of Ca-P-coated Mg alloy.

### 4.2. Morphological effect of OCP and HAp coatings on cell adhesion and proliferation

The initial density of MG-63 cells adhered on OCP-AZ31 was slightly lower than that on HAp-AZ31, although no statistically significant difference was observed (Figures 4 and 6). Cell elongation and proliferation on OCP-AZ31 was suppressed compared with those on HAp-AZ31 (Figures 4, 6 and 8). Focal contacts were formed on crystal tips, which were more widely spaced on the OCP coating than on the HAp coating.

Holthaus et al. [31] reported that osteoblast cells formed focal contacts on top of HAp micropatterns, preferentially toward the inside (valley floor), when the width of the micropattern was less than 40 μm^31^. Choi et al. [46] reported that the elongation of human foreskin fibroblasts was significantly suppressed on needle-like nanopillars of silicon with 500–600 nm height and a 230 nm spacing, with most filopodia extending along the sharp edges of blade-like nanogrates^46^. Fibroblasts seeded on nanopillars with 50–100 nm height exhibited good filopodial extension. Chua et al. [47] reported that murine neural progenitor cells adhered preferentially on the raised region of micro-gratings with a 2 μm spacing and 2–4 μm depth because neurites could not bend to reach the bottom of deep grooves (2–4 μm).^47^ These facts indicate that the heights of OCP plate-like and HAp rod-like crystals, which were 2–5 μm, were too high for pseudopodia of osteoblast-like cells to reach the bottom. Thus, focal adhesions were formed only on tips of plate-like and rod-like crystals in this study. In addition, the spacing between tips of OCP plate-like crystals, which was 0.8–1.1 μm, was too wide for osteoblast-like cells to extend pseudopodia to the next crystal tip. The spacing between tips of HAp rod-like crystals, which was 0.2–0.3 μm, did not hinder the extension of pseudopodia. The suppression of cell elongation and proliferation on OCP-AZ31 is thus attributed to the sparse formation of focal contacts.

In addition to the above topographic explanation, chemical properties depending on the crystal structure of OCP and HAp might also have influenced the adhesion behavior of cells. Hexagonal HAp crystals have two major crystal planes, the *a*,*b*-plane and the *c*-plane. The *a*,*b*-plane is enriched with calcium ions and is thus positively charged, while the *c*-plane is enriched with phosphate and hydroxide ions and is negatively charged. Kizuki et al. [48] reported that Ca-P deposition and MC3T3-E1 cell adhesion were enhanced on the negatively charged HAp surface compared with those on a positively charged surface.^48^ Zhuang et al. [49] reported that the initial adhesion of MC3T3-E1 cells on HAp ceramics depended on the preferential orientation of the *a*- and *b*-planes to the surface and the cell-attachment efficiency decreased with increasing surface orientation degree of *a*- and *b*-planes.^49^ Therefore, the negative charge of the *c*-plane possibly enhanced focal contact formation on the tips of rod-like HAp crystals and ridges of plate-like OCP crystals.

## 5. Conclusions

Cell adhesion and proliferation behavior were examined on OCP and HAp coatings formed on AZ31 using human osteosarcoma MG-63 cells. HAp and OCP coatings improved cell viability by preventing corrosion of the substrate AZ31. The surface pH of the HAp coating was slightly higher than that of the OCP coating, and both coatings suppressed H_2_O_2_ generation; however, further investigation is necessary to understand the effect of such surface chemical properties on cell behavior. HAp coating remarkably improved cell adhesion and proliferation, and showed a dense and homogeneous distribution of living cells, while dead cells were localized on/over corrosion pits of the substrate. OCP coating showed a similar initial cell adhesion density on the HAp coating; however, cell elongation and subsequent cell proliferation were reduced, leading to sparsely distributed living and dead cells. Cells formed the focal contacts on the tips of rod-like HAp and plate-like OCP crystals. The focal contacts on HAp coating with narrower tip spacing showed higher density than those on OCP coating. Consequently, the shape, orientation and density of Ca-P crystals in the coatings have the greatest influence on the spacing of the focal contacts that governs cell adhesion, elongation and proliferation; a higher density of focal contacts leads to increased cell proliferation. In addition, the local cell death on HAp-AZ31 indicates the importance of entire surface observation for the evaluation of cell compatibility.

## Disclosure statement

No potential conflict of interest was reported by the authors.

## Funding

This work was supported by World Premier International Research Centre Initiative (WPI) on Materials Nanoarchitectonics (MANA), the Ministry of Education, Culture, Sports, Science and Technology (MEXT) and NIMS Molecule and Material Synthesis Platform in ‘Nanotechnology Platform Project’ operated by MEXT. In addition, this study was partially supported by Grant-in-Aid for Scientific Research (C) [grant number 25420742] and Grant-in-Aid for Scientific Research (B) [grant number 16H04511] from Japan Society for the Promotion of Science (JSPS).

## Supplemental data

The supplemental material for this paper is available online at http://dx.doi.org/10.1080/14686996.2016.1266238

## Supplementary Material

supplemental_material_161124.docx

## References

[CIT0001] Heublein B, Rohde R, Kaese V (2003). Biocorrosion of magnesium alloys: a new principle in cardiovascular implant technology?. Heart.

[CIT0002] Staiger MP, Pietak AM, Huadmai J (2006). Magnesium and its alloys as orthopedic biomaterials: a review. Biomaterials.

[CIT0003] Witte F, Calliess T, Windhagen H (2008). Biodegradable synthetic implant materials. clinical applications and immunological aspects. Orthopade.

[CIT0004] Zeng RC, Dietzel W, Witte F (2008). Progress and challenge for magnesium alloys as biomaterials. Adv Eng Mater.

[CIT0005] Witte F (2010). The history of biodegradable magnesium implants: a review. Acta Biomater.

[CIT0006] Witte F, Feyerabend F, Maier P (2007). Biodegradable magnesium–hydroxyapatite metal matrix composites. Biomaterials.

[CIT0007] Waizy H, Seitz JM, Reifenrath J (2013). Biodegradable magnesium implants for orthopedic applications. J Mater Sci.

[CIT0008] Yamasaki Y, Yoshida Y, Okazaki M (2003). Action of FGMgCO3Ap-collagen composite in promoting bone formation. Biomaterials.

[CIT0009] Bussière FI, Gueux E, Rock E (2002). Increased phagocytosis and production of reactive oxygen species by neutrophils during magnesium deficiency in rats and inhibition by high magnesium concentration. Br J Nutr.

[CIT0010] Kuwahara H, Al-Abdullat Y, Mazaki N (2001). Precipitation of magnesium apatite on pure magnesium surface during immersing in Hank’s solution. Mater Trans.

[CIT0011] Al-Abdullat Y, Tsutsumi S, Nakajima N (2001). Surface modification of magnesium by NaHCO3 and corrosion behavior in Hank's solution for new biomaterial applications. Mater Trans.

[CIT0012] Shadanbaz S, Dias GJ (2012). Calcium phosphate coatings on magnesium alloys for biomedical applications: a review. Acta Biomater.

[CIT0013] Hornberger H, Virtanen S, Boccaccini AR (2012). Biomedical coatings on magnesium alloys – a review. Acta Biomater.

[CIT0014] Li LC, Gao JC, Wang Y (2004). Evaluation of cyto-toxicity and corrosion behavior of alkali-heat-treated magnesium in simulated body fluid. Surf Coat Tech.

[CIT0015] Li N, Zheng YF (2013). Novel magnesium alloys developed for biomedical application: a review. J Mater Sci Technol.

[CIT0016] Xu LP, Pan F, Yu GN (2009). In vitro and in vivo evaluation of the surface bioactivity of a calcium phosphate coated magnesium alloy. Biomaterials.

[CIT0017] Geng F, Tan L, Zhang BC (2009). Study on B-Tcp coated porous mg as a bone tissue engineering scaffold material. J Mater Sci Technol.

[CIT0018] Wong HM, Yeung KWK, Lam KO (2010). A biodegradable polymer-based coating to control the performance of magnesium alloy orthopaedic implants. Biomaterials.

[CIT0019] Gopi D, Murugan N, Ramya S (2014). Electrodeposition of a porous strontium-substituted hydroxyapatite/zinc oxide duplex layer on AZ91 magnesium alloy for orthopedic applications. J Mater Chem B.

[CIT0020] Zhang M, Cai S, Shen SB (2016). In-situ defect repairing in hydroxyapatite/phytic acid hybrid coatings on AZ31 magnesium alloy by hydrothermal treatment. J Alloy Compd.

[CIT0021] Song YW, Shan DY, Han EH (2008). Electrodeposition of hydroxyapatite coating on AZ91D magnesium alloy for biomaterial application. Mater Lett.

[CIT0022] Wen CL, Guan SK, Peng L (2009). Characterization and degradation behavior of AZ31 alloy surface modified by bone-like hydroxyapatite for implant applications. Appl Surf Sci.

[CIT0023] Chai H, Guo L, Wang XT (2012). In Vitro and in Vivo evaluations on osteogenesis and biodegradability of a Ss-tricalcium phosphate coated magnesium alloy. J. Biomed. Mater. Res. A.

[CIT0024] Zhang YJ, Zhang GZ, Wei M (2009). Controlling the biodegradation rate of magnesium using biomimetic apatite coating. J Biomed Mater Res B.

[CIT0025] Leon B, Jansen JA (2009). Thin calcium phosphate coatings for medical implants.

[CIT0026] Hiromoto S, Shishido T, Yamamoto A (2008). Precipitation control of calcium phosphate on pure magnesium by anodization. Corros Sci.

[CIT0027] Bigi A, Falini G, Foresti E (1993). Magnesium influence on hydroxyapatite crystallization. J Inorg Biochem.

[CIT0028] Fadeev IV, Shvorneva LI, Barinov SM (2003). Synthesis and Structure of Magnesium-Substituted Hydroxyapatite. Inorg Mater.

[CIT0029] Ioku K (1996). Hydroxyapatite and related calcium phosphates as ceramic biomaterials. Inorg Mater.

[CIT0030] Kamitakahara M, Uno Y, Ioku K (2014). Behavior of osteoblast-like cells on calcium-deficient hydroxyapatite ceramics composed of particles with different shapes and sizes. J Mater Sci: Mater Med.

[CIT0031] Holthaus MG, Stolle J, Treccani L (2012). Orientation of human osteoblasts on hydroxyapatite-based microchannels. Acta Biomater.

[CIT0032] Hiromoto S (2009). High corrosion resistance of magnesium coated with hydroxyapatite directly synthesized in an aqueous solution. Electrochim Acta.

[CIT0033] Hiromoto S, Tomozawa M (2010). Corrosion Behavior of magnesium with hydroxyapatite coatings formed by hydrothermal treatment. Mater Trans.

[CIT0034] Tomozawa M, Hiromoto S, Harada Y (2010). Microstructure of hydroxyapatite-coated magnesium prepared in aqueous solution. Surf Coat Tech.

[CIT0035] Tomozawa M, Hiromoto S (2011). Microstructure of hydroxyapatite- and octacalcium phosphate-coatings formed on magnesium by a hydrothermal treatment at various pH values. Acta Mater.

[CIT0036] Hiromoto S, Tomozawa M (2011). Hydroxyapatite coating of AZ31 magnesium alloy by a solution treatment and its corrosion behavior in NaCl solution. Surf Coat Tech.

[CIT0037] Ohtsu N, Hiromoto S, Yamane M (2013). Chemical and crystallographic characterizations of hydroxyapatite- and octacalcium phosphate-coatings on magnesium synthesized by chemical solution deposition using XPS and XRD. Surf Coat Tech.

[CIT0038] Hiromoto S, Tomozawa M, Maruyama N (2013). Fatigue property of a bioabsorbable magnesium alloy with a hydroxyapatite coating formed by a chemical solution deposition. J Mech Behav Biomed Mater.

[CIT0039] Hiromoto S, Inoue M, Taguchi T (2015). In vitro and in vivo biocompatibility and corrosion behaviour of a bioabsorbable magnesium alloy coated with octacalcium phosphate and hydroxyapatite. Acta Biomater.

[CIT0040] Curtis A, Wilkinson C (1997). Topographical control of cells. Biomaterials.

[CIT0041] Mann CK, Yoe JH (1956). Spectrophotometric determination of magnesium with sodium 1-Azo-2-hydroxy-3-(2,4-dimethylcarboxanilido)-naphthalene-1'-(2-hydroxybenzene-5-sulfonate). Anal Chem.

[CIT0042] Watanabe H, Tanaka H (1977). Dual-Wavelength spectrophotometric determination of magnesium with xylidyl blue I and nonionic surfactant. Bunseki-kagaku.

[CIT0043] Sawai J, Kojima H, Shimizu K (1997). Inorganic antibacterial agents. Inorg Mater.

[CIT0044] Kim J, Gilbert JL (2016). Cytotoxic effect of galvanically coupled magnesium–titanium particles. Acta Biomater.

[CIT0045] Seuss F, Seuss S, Turhan MC (2011). Corrosion of Mg alloy AZ91D in the presence of living cells. J Biomed Mater Res B.

[CIT0046] Choi C-H, Hagvall SH, Wu BM (2007). Cell interaction with three-dimensional sharp-tip nanotopography. Biomaterials.

[CIT0047] Chua JS, Chng C-P, Moe AAK (2014). Extending neurites sense the depth of the underlying topography during neuronal differentiation and contact guidance. Biomaterials.

[CIT0048] Kizuki T, Ohgaki M, Katsura M (2003). Effect of bone-like layer growth from culture medium on adherence of osteoblast-like cells. Biomaterials.

[CIT0049] Zhuang Z, Fujimi TJ, Nakamura M (2013). Developement of a, B-plane-oriented hydroxyapatite ceramics as models for living bones and their cell adhesion behavior. Acta Biomater.

