# Supplementary material for: Micromorphological effect of calcium phosphate coating on compatibility of magnesium alloy with osteoblast
Source: Sci Technol Adv Mater. 2017 Jan 23;18(1):96–109. doi: 10.1080/14686996.2016.1266238 (PMC5259964; doi:10.1080/14686996.2016.1266238)
Supplement: supplemental_material_161124.docx [file tsta_a_1266238_sm0276.docx]

**Supplemental material**

**Micromorphological effect of calcium phosphate coating on compatibility of magnesium alloy with osteoblast**

Sachiko Hiromoto^1*^ and Tomohiko Yamazaki^2^

*^1^ Research Center for Structural Materials, National Institute for Materials Science, Tsukuba, Japan*

*^2^ Research Center for Functional Materials, National Institute for Materials Science, Tsukuba, Japan*

**1. Materials and methods**

**1.1. Cross section observation of OCP and HAp coatings**

Cross-section specimens were prepared by scraping off the coatings from OCP- and HAp-AZ31. Back scattered electron (BSE) images were observed using SEM (FEI Quanta FEG250, USA) for the same field of view of Figs. 3(c) and 3(d).

**1.2. Implantation test in mouse**

Octacalcium phosphate (OCP) and hydroxyapatite (HAp) coatings were formed on AZ31 disk (8 mm^φ^ x 1 mm^t^) in the same procedure except the treatment period was 4 hours for the implantation test. The OCP- and HAp-AZ31 discs were implanted subcutaneously into the back of NF-κB/luciferase transgenic mice (BALB/C-Tg (NF-κB-REluc)-Xen, Xenogen, Alameda, CA). Five mice were used for each OCP- and HAp-AZ31. Details of implantation procedure were described in the previous report [1]. After 16 weeks of implantation, the discs were removed from the mice and the surface was characterized using a backscattering electron microscope (BSEM; Miniscope TM3000, Hitachi, Japan) and X-ray diffraction (XRD; RINT-Ultima III, Rigaku, Japan). The soft tissues around the implanted discs were fixed in 10% neutral buffered formalin, embedded in paraffin, sectioned at 5 μm thickness and stained with hematoxylin and eosin (HE). Thickness of fibrous layer formed around the implanted discs was obtained from the HE-stained cross-sectional images. The results were compared using Student’s *t*-test and statistical significance was considered when the *p* value was less than 0.05.

**2. Results and discussion**

**2.1. Cross section observation of OCP and HAp coatings**

Supplemental Figure 1 shows cross-section backscattered electron images of OCP and HAp coatings. Composition difference emphasizes the boundary between inner and outer layers comparing with the topographic difference on SEM images in Figs. 3(c) and 3(d). In the inner layer of HAp coating, original primary particles of dome shape were easily recognized. Rod-like HAp crystals grew from each dome.


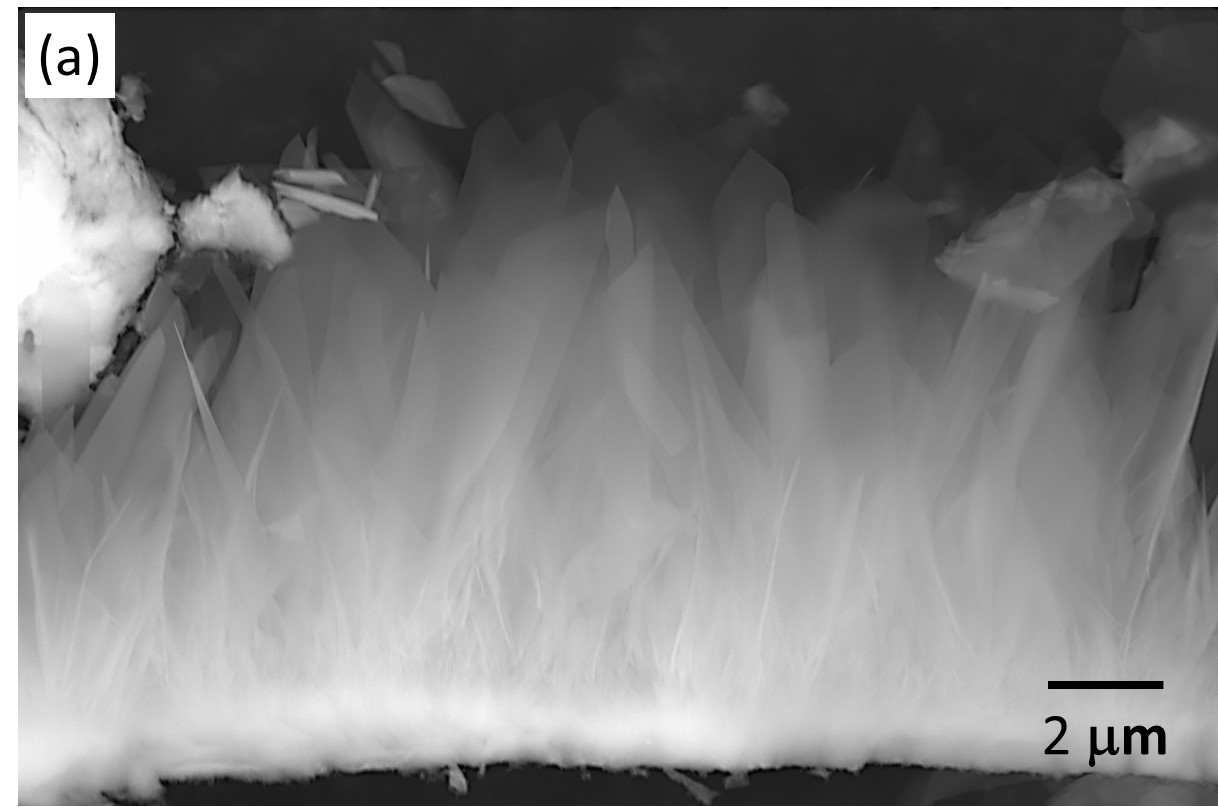


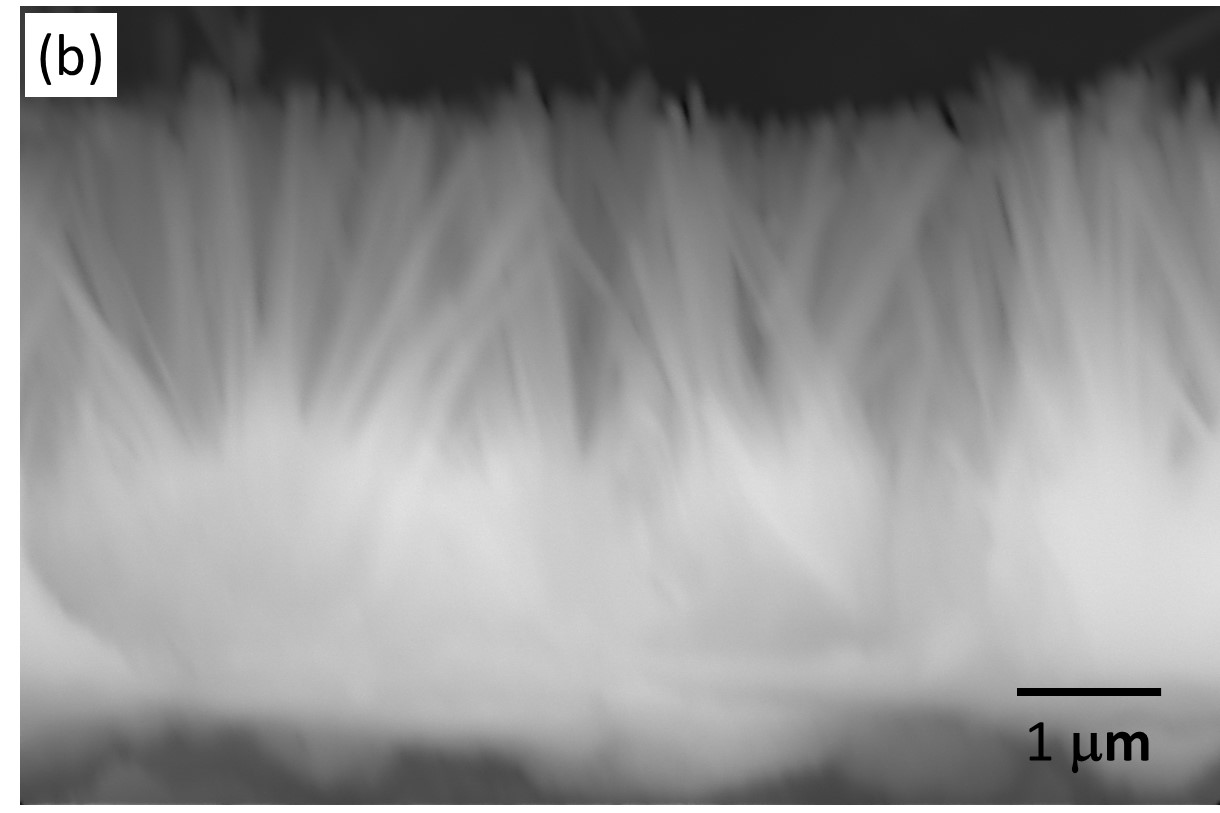


Suppl-Fig. 1 Cross-sectional backscattered electron images of (a) OCP and (b) HAp coatings.

**2.2. Host response to OCP- and HAp-AZ31**

Supplemental Figure 2 shows cross-sectional images of the HE-stained tissues which contacted to the implanted OCP- and HAp-AZ31 [1]. A fibrous tissue layer was formed around the implanted specimens. The layer thickness corresponds approximately to the magnitude of foreign body response. Average of the layer thickness around 3 samples is shown in Suppl-Fig. 2(c). Although statistically significant difference was not observed in the fibrous layer thickness between OCP- and HAp-AZ31, the thickness around OCP-AZ31 was slightly larger than that around HAp-AZ31. Supplemental Figure 3 shows surface BSEM image of OCP- and HAp-AZ31 as-prepared and implanted subcutaneously for 16 weeks [1]. Almost all outer plate-like OCP crystals were dissolved and a part of rod-like HAp crystals was disappeared in small areas of several micrometers.


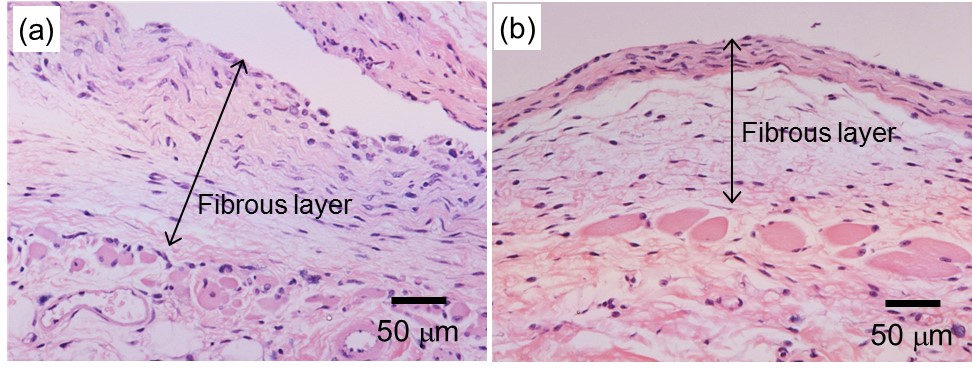

Suppl-Fig. 2 Cross-sectional images of HE-stained tissues which contacted to implanted (a) OCP- and (b) HAp-AZ31 [1]. (c) Thickness of fibrous tissue layer formed around implanted OCP- and HAp-AZ31. n.s.: not significant.

The plate-like OCP crystals, which limited the cell proliferation of human osteosarcoma cells MG-63 (Figs. 4 and 6), almost disappeared in vivo for 16 weeks. This fact suggests that the effect of micromorphology of OCP coating might decrease with disappearing of plate-like crystals. As the result, no statistically significant difference in the thickness of fibrous tissue layer was observed between OCP- and HAp-AZ31. However, a slight thicker fibrous tissue layer around OCP-AZ31 than HAp-AZ31 might be able to attributed to the less cell adhesion property of OCP-AZ31 than HAp-AZ31.

To understand the in vivo effect of the micromorphology of OCP and HAp coatings, further investigation is necessary and a short-period implantation test would be appropriate.


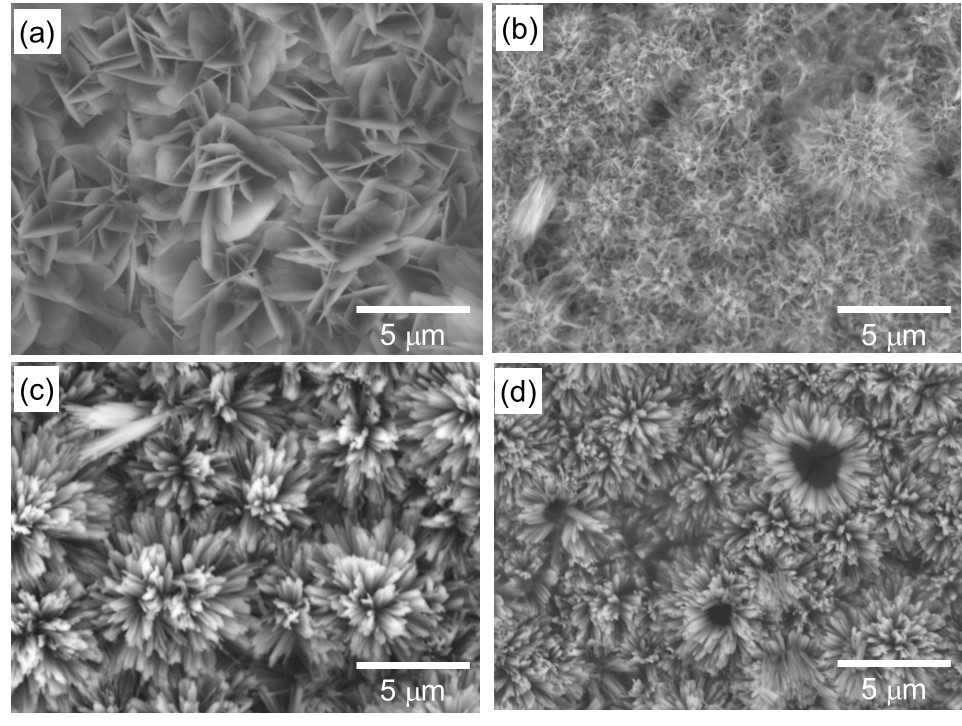


Suppl-Fig. 3 Surface BSEM images of (a) as-prepared OCP- and (b) 16 weeks-implanted OCP-, (c) as-prepared HAp- and (d) 16 weeks-implanted HAp-AZ31 [1].

**Reference**

[1] Hiromoto S, Inoue M, Taguchi T, et al. In Vitro and in Vivo Biocompatibility and Corrosion Behaviour of a Bioabsorbable Magnesium Alloy Coated with Octacalcium Phosphate and Hydroxyapatite. Acta Biomater. 2015; 11: 520-530.
